# Patient satisfaction with pharmaceutical services in the context of chief pharmacist system: a comparative study from Shaanxi Province, China

**DOI:** 10.3389/fpubh.2026.1741020

**Published:** 2026-02-10

**Authors:** Yongjian Xu, Hui Li, Yazhuo Liu, Jia Wu, Jiaxin Sun, Xueyan Han, Shi An, Yinjun Tan, Yunlong Song, Yongbing Cheng, Junteng Hu

**Affiliations:** 1 School of Public Policy and Administration, Xi’an Jiaotong University, Xi’an, China; 2 Shaanxi Provincial Centre for Disease Control and Prevention, Xi’an, China; 3 Institute of Science and Technology, Xi’an Jiaotong University, Xi’an, China

**Keywords:** chief pharmacist system, China, comparative study, patient satisfaction, pharmaceutical services

## Abstract

**Background:**

To improve rational use of medicines for better pharmaceutical services, the Chinese government has piloted Chief Pharmacist System (CPS). This study aims to explore the relationship between CPS and patient satisfaction by comparing satisfaction with pharmaceutical services between pilot and non-pilot public hospitals.

**Methods:**

A cross-sectional survey was conducted between July and August 2022. A total of 492 patients were included, with 276 from 9 pilot hospitals and 216 from 9 non-pilot hospitals. Patient satisfaction was measured using a 22-item questionnaire designed by Khudair and Raza, and a 0–100 scale overall satisfaction rating question. Multilevel linear regression models with random intercepts were used to account for the clustering of patients within hospitals to analyze the determinants of patient satisfaction.

**Results:**

The mean score of overall patient satisfaction in the pilot hospitals (89.13 ± 10.99) was higher than that of the non-pilot hospitals (85.67 ± 13.87) (*p* = 0.002). Analysis by dimension showed that patients in the pilot hospitals as compared with those in non-pilot hospitals showed significantly higher percentages of ‘satisfied’ in promptness of medication receipt (87.32% vs. 74.54%), pharmacists’ attitude of solving issues (88.04% vs. 75.93%), understanding cases of pharmacist (77.90% vs. 64.35%), clarity of medication label (77.54% vs. 63.89%), cleanliness and acceptability of pharmacy area (87.32% vs. 79.63%), working hours of pharmacy (80.80% vs. 71.76%), and medication education (all items except for medication dosage guidance). These differences were statistically significant (*p* < 0.05). While the proportion of patients satisfied with medication education time was the lowest (30.43%) compared to other items, the pilot group still performed better than the non-pilot group. Multilevel analysis showed that the implementation of CPS was positively associated with patient satisfaction (*β* = 3.264, *p* = 0.010).

**Conclusion:**

Patient satisfaction with pharmaceutical services was significantly higher in pilot hospitals than in non-pilot hospitals. The implementation of CPS was positively associated with patient satisfaction with pharmaceutical services. Policymakers could consider the broader adoption of CPS as a beneficial strategy for enhancing patient satisfaction. This could be complemented by efforts to ensure sufficient medication education, optimize pharmacy physical spaces, and advance targeted pharmacist training.

## 1 Introduction

Irrational use of medicines remains one of the pressing issues in many healthcare systems across the world (1). This results in widespread wastage of scarce resources and health hazards (1, 2). In 2017, the World Health Organization (WHO) highlighted that the global cost related to medication errors was estimated at US$42 billion annually (3). Moreover, at least 1 in 20 patients were affected by preventable medication-related harm globally (4). These issues were particularly acute in contexts where traditional pharmacy administration was limited. In China, the primary responsibility of hospital pharmacy departments was largely restricted to medication distribution, which hindered interdisciplinary collaboration with hospital administration (5, 6). Furthermore, pharmacy leaders were tasked mainly with ensuring drug supply but lacked the institutional authority to implement clinical pharmaceutical management (7–9). Based on this, the Chief Pharmacist System (CPS) was proposed by the Chinese government in 2016 to improve the quality of pharmaceutical administration and services.

In the context of CPS, the pharmacy department has been transformed into an administrative and technical department, focusing on high-quality pharmacy services (10). The chief pharmacist is defined as the organizer and leader of the pharmacy administration at the hospital, who is responsible for building collaborative relationships among different departments, controlling medication procurement budget, promoting pharmacists’ development, and advancing the pharmacy discipline (11). Furthermore, CPS mainly includes three key strategies. Firstly, an indicator system for the rational use of medicines is established, whereby clinical departments meeting these indicators are allocated financial incentives. Secondly, pharmacy departments are entailed to transition pharmacy operations from a “drug-centered” model to a “patient-centered” one, where pharmacists are actively engaged in clinical pharmaceutical services. Thirdly, an interdisciplinary team is established to monitor and review key medicines, such as ancillary medicines, high-priced medicines, off-label use medicines, and those with abnormal usage surges.

Previous studies showed that medicine revenues and expenditures changed after the implementation of CPS. A study found that CPS could achieve substantial reductions in medicine expenditures and significant promotion of rational medicine use (5). Another indicated that the proportion of outpatient herbal medicine revenues in healthcare settings increased by 11.9% (12). As the CPS remains in the exploratory phase, it inevitably faces numerous challenges, including an unclear delineation of authority between the chief pharmacist and the pharmacy department director, as well as a lack of clinical capabilities among pharmacists (10). However, these studies had mainly focused on the perspectives of both pharmacy managers and pharmacists (5, 10, 12), leaving the patient perspective underexplored. As a direct manifestation of service recipients’ perceptions, patient satisfaction is a fundamental component in the assessment of policy efficacy (13, 14). It reflects the extent to which the services delivered by the policy meet patients’ needs, expectations, and preferences (15). Therefore, analyzing patient satisfaction within the CPS context to identify strengths and weaknesses in service performance offers critical evidence for improving healthcare management and public health policy (16, 17). To the best of our knowledge, there remains a limitation in empirical analysis regarding the association between the CPS and patient satisfaction with pharmaceutical services.

A cross-sectional survey was designed to investigate the current state of patient satisfaction with pharmaceutical services in public hospitals that had piloted CPS and those that had not. The study aims to explore the association between CPS and patient satisfaction by comparing pilot and non-pilot public hospitals. The study hypothesized that patients in CPS pilot hospitals would report significantly higher satisfaction than those in non-pilot hospitals. This study will provide a reference for governments to advance CPS implementation, as well as for guiding hospitals to adopt pharmacist incentives for better patient satisfaction with pharmaceutical services. In addition, this study will contribute to readers’ understanding of CPS in China.

## 2 Materials and methods

### 2.1 Study design and setting

As one of the pioneering pilot regions for CPS in China, Shaanxi Province initiated CPS in 10 secondary hospitals and above in September 2017. This cross-sectional survey was conducted in pilot and non-pilot public hospitals in Shaanxi Province. The pilot group was defined as a public hospital that had fully implemented CPS for at least 1 year prior to the survey. The non-pilot group was defined as a public hospital that had not implemented CPS. The survey was performed between July and August 2022.

### 2.2 Data sources and sample selection

Multistage stratified random sampling was adopted to select participants in this study. First, based on socioeconomic development levels, Shaanxi province was stratified into three regions: Northern Shaanxi, Southern Shaanxi, and Guanzhong. Second, three pilot hospitals (including two tertiary and one secondary hospital) were randomly selected from each region. Subsequently, based on the criteria of geographic location, institutional grade, type, and scale, a panel of experts selected nine non-pilot hospitals to serve as one-to-one matched controls for the pilot hospitals. The pilot and non-pilot groups were confirmed to meet the homogeneity assumption in their baseline characteristics. Third, the minimum sample size was calculated using Fisher’s formula for a proportion: $\;n=P\times (1-P)\times Z_{\alpha /2}^{2}/d^{2}$ (18). We used a conservative estimate of 50% for patient satisfaction with pharmaceutical services ( P = 0.50) which maximizes variance to cover all potential satisfaction rates, along with a confidence level of 0.95 ( $Z_{\alpha /2}=$ 1.96) and a desired precision ( d ) of 0.05. A minimum sample size of 385 was determined based on this equation. To account for potential non-response and to increase statistical power, the sample size was increased by approximately one-third to 512 (19). Then, this number was allocated proportionally to each hospital based on their institutional scale. Within each participating hospital, participants were randomly selected from daily inpatient pharmacy dispensing records throughout the survey period. After excluding 20 with incomplete responses, 492 valid questionnaires were retained, yielding an effective response rate of 96%. This sample size exceeded the calculated minimum requirement. The final sample consisted of 276 patients from pilot hospitals and 216 from non-pilot hospitals.

### 2.3 Inclusion and exclusion criteria

Participants were recruited from inpatients receiving pharmaceutical services at the participating hospitals. Patients who had incomplete pharmacy records, a critical illness, or cognitive impairment were excluded. All participants provided written informed consent prior to enrolling in the survey.

### 2.4 Ethics approval

The study protocol has been approved by the Ethics Committee of The First Affiliated Hospital of Xi’an Jiaotong University (Approval Number: LLSBPJ-2024-WT-016). All respondents were asked for their consent before participation in the study.

### 2.5 Measurement

The dependent variable was patient satisfaction with pharmaceutical services, measured using the 22-item questionnaire developed by Khudair and Raza (20), which was originally constructed to assess outpatient satisfaction with pharmacy services in public hospitals. It has demonstrated excellent internal consistency (Cronbach’s *α* = 0.941) in previous studies (20, 21). In this study, the English version was translated into Chinese following a forward-backward translation procedure (22), which was conducted by two independent bilingual authors (XYJ and HJT) with academic backgrounds in health policy and management. To verify its face and content validity, the translated questionnaire was reviewed and revised by two independent experts in pharmacy services. Prior to the main survey, the questionnaire was pre-tested on 50 patients to assess its clarity, relevance, and cultural appropriateness. The Chinese version of the questionnaire also demonstrated high reliability, with a Cronbach’s alpha of 0.924. The Kaiser-Meyer-Olkin measure (KMO = 0.918) and Bartlett’s test of sphericity ( χ2 = 6744.884, P < 0.001) indicated that the data were suitable for factor analysis (23). Factor analysis revealed that the 22 items of the questionnaire loaded on five factors, accounting for 78.31% of the total variance. The factor loading pattern supported a stable five-factor structure for the scale. The questionnaire comprises 22 items across the five dimensions, including service promptness, pharmacist attitude, medication supply, pharmacy location, and medication education. All items are positively phrased and rated on a 5-point Likert scale, ranging from “strongly disagree” (1 point) to “strongly agree” (5 points). Responses were dichotomized into “dissatisfied” (scores 1–3) and “satisfied” (scores 4–5), which was consistent with previous studies on pharmaceutical services (24, 25). Collapsing a 5-point scale into a dichotomous scale is an effective approach to reducing ambiguous mid-point responses and enhancing the clarity and actionability of the data for quality improvement purposes in clinical settings, such as hospitals (26). Additionally, respondents were asked to score their overall satisfaction with pharmacy services on a scale from 0 to 100, with higher scores indicating greater satisfaction.

The independent variable was the Chief Pharmacist System (CPS). This binary variable was coded as “Implemented” if the participant’s treating hospital had piloted the CPS for at least 1 year, and “Not Implemented” if the hospital had not piloted the policy. In addition, based on previous empirical studies, sociodemographic characteristics that may be associated with patient satisfaction were considered in the study (9, 13, 14). Therefore, the following control variables were included in the analysis: age (≤34 years old, 35–60 years old, ≥61 years old), gender (male, female), annual income (≤30,000 CNY, 30,001–60,000 CNY, ≥60,001 CNY), marital status (unmarried, married), education level (elementary and below, junior high school, high school and above), area (Southern Shaanxi, Northern Shaanxi, Guanzhong), residence (rural, urban), hospital grade (tertiary hospital, secondary hospital), department (internal medicine, surgery, others), and the attending physician’s professional title (senior title, non-senior title).

### 2.6 Data analysis

Categorical variables were described using the number and percentage. The overall satisfaction score with pharmaceutical services was reported as the mean and standard deviation (SD). Violin plots were used to demonstrate the distribution of overall satisfaction scores between the pilot and non-pilot groups. The chi-square test was performed to compare the difference between the pilot and non-pilot groups in baseline characteristics. One-way analysis of variance (ANOVA) and independent samples *t*-tests were used to assess the difference in overall patient satisfaction with pharmaceutical services score among groups.

In multilevel models for cross-sectional data, individuals (level 1) are nested within institutions (level 2) (27, 28). The basic operational steps of multilevel model establishment are as follows: First, a null model (random intercept null model) was established to examine the hierarchical structure of the data and to calculate the intraclass correlation coefficient (ICC). Second, explanatory variables are introduced as fixed effects to extend the null model, allowing evaluation of the significance of predictors at different levels.

The ICC represents the proportion of the total variance that is attributable to differences between groups, calculated as the ratio of the between-group variance to the total variance. The formula for the ICC is as follows:


$$\mathrm{ICC}=(\frac{\sigma_{u}^{2}}{\sigma_{u}^{2}+\sigma_{\varepsilon }^{2}})$$

$\sigma_{u}^{2}$ presents the between-institution variance and $\sigma_{\varepsilon }^{2}$ presents the within-institution variance.

The null model is specified as follows:

Level 1 (individual-level model):


$$Y_{\mathrm{ij}}=\beta_{0j}+\varepsilon_{\mathrm{ij}}$$

Where i represents the i -th individual. j represents the j -th institution. $Y_{\mathrm{ij}}$ is the value of the dependent variable for the i -th individual in the j -th institution. $\beta_{0j}$ is the intercept for the j -th institution. $\varepsilon_{\mathrm{ij}}\;$ is the individual-level random error, which is assumed to be normally distributed with a mean of 0 and variance $\sigma_{\varepsilon }^{2}$ .

Level 2 (institutional-level model):


$$\beta_{0j}=\gamma_{00}+u_{0j}$$

Where $\gamma_{00}$ is the grand mean of the dependent variable. $u_{0j}$ is the institution-specific random effect, representing the deviation of institution $j^{’}$ s mean from the grand mean. It is assumed to be normally distributed with a mean of 0 and variance $\sigma_{u}^{2}$ .

The results from the null model showed that the between-institution variance was 2.854. The ICC was 0.0186 and was statistically significant (*p* < 0.05), indicating that a small but non-negligible portion (1.86%) of the total variance in satisfaction scores is attributable to between-institution differences (29). While the magnitude of clustering is limited, the significant ICC justifies the use of a multilevel modeling approach to account for this hierarchical data structure and to obtain robust estimates.

The multilevel random intercept model with an independent variable can be written as follows:


$$Y_{\mathrm{ij}}=\gamma_{00}+\beta_{1j}x_{1\mathrm{ij}}+(u_{0j}+\varepsilon_{\mathrm{ij}})$$

Where $\beta_{1j}$ is unknown coefficients to be estimated.

The model introduces predictors at both levels while allowing the intercept to vary randomly across institutions. Model fit was assessed by comparing Akaike’s Information Criterion (AIC) and Bayesian Information Criterion (BIC) between the null and full models. The decrease in these indices indicated an improved model fit after the inclusion of the explanatory variables (30). All analyses were conducted using the Stata 17.0 software, with statistical significance indicated by P < 0.05.

## 3 Results

### 3.1 Basic characteristics

The basic characteristics of sample are shown in Table 1. Of 492 participants, 50.81% were males, 47.76% were 34 years old or younger, and 54.47% were from rural areas. The vast majority (93.29%) had an annual income of 60,000 CNY or below. More than half of patients had attained a high school education or higher, 88.41% were married, and nearly two-thirds of the patients were from tertiary hospitals. Moreover, comparing patients from pilot group and non-pilot group, there were more males (52.90% vs. 48.15%) and rural participants (58.70% vs. 49.07%) in the former group.

**Table 1:** Basic characteristics of sample and comparisons between the non-pilot and pilot groups, n (%).

Variables	Total	Non-pilot group	Pilot group	χ^2^	*P*
Gender				1.094	0.296
Male	250(50.81)	104(48.15)	146(52.90)		
Female	242(49.19)	112(51.85)	130(47.10)		
Age (years)				1.790	0.409
≤34	235(47.76)	96(44.44)	139(50.36)		
35–60	156(31.71)	74(34.26)	82(29.71)		
≥61	101(20.53)	46(21.30)	55(19.93)		
Income (CNY)^α^				0.469	0.791
≤30,000	203(41.26)	92(42.59)	111(40.22)		
30,001-60,000	256(52.03)	111(51.39)	145(52.54)		
≥60,001	33(6.71)	13(6.02)	20(7.25)		
Marital status				0.085	0.771
Unmarried	57(11.59)	24(11.11)	33(11.96)		
Married	435(88.41)	192(88.89)	243(88.04)		
Education level				1.578	0.454
Elementary or below	46(9.35)	20(9.26)	26(9.42)		
Junior high school	181(36.79)	86(39.81)	95(34.42)		
High school or above	265(53.86)	110(50.93)	155(56.16)		
Area				6.478	0.039
Southern Shaanxi	71(14.43)	40(18.52)	31(11.23)		
Northern Shaanxi	166(33.74)	75(34.72)	91(32.97)		
Guanzhong	255(51.83)	101(46.76)	154(55.80)		
Residence				4.523	0.033
Urban	224(45.53)	110(50.93)	114(41.30)		
Rural	268(54.47)	106(49.07)	162(58.70)		
Department				2.466	0.291
Internal medicine	214(43.50)	91(42.13)	123(44.57)		
Surgery	158(32.11)	65(30.09)	93(33.70)		
Others	120(24.39)	60(27.78)	60(21.74)		
Professional title				0.001	0.967
Senior title	307(62.40)	135(62.50)	172(62.32)		
Non-senior title	185(37.60)	81(37.50)	104(37.68)		
Hospital grade				0.001	0.981
Tertiary	330(67.07)	145(67.13)	185(67.03)		
Secondary	162(32.93)	71(32.87)	91(32.97)		

^α^Income: Annual income in Chinese yuan (CNY).

### 3.2 Overall patient satisfaction with pharmaceutical services

**Figure 1:**
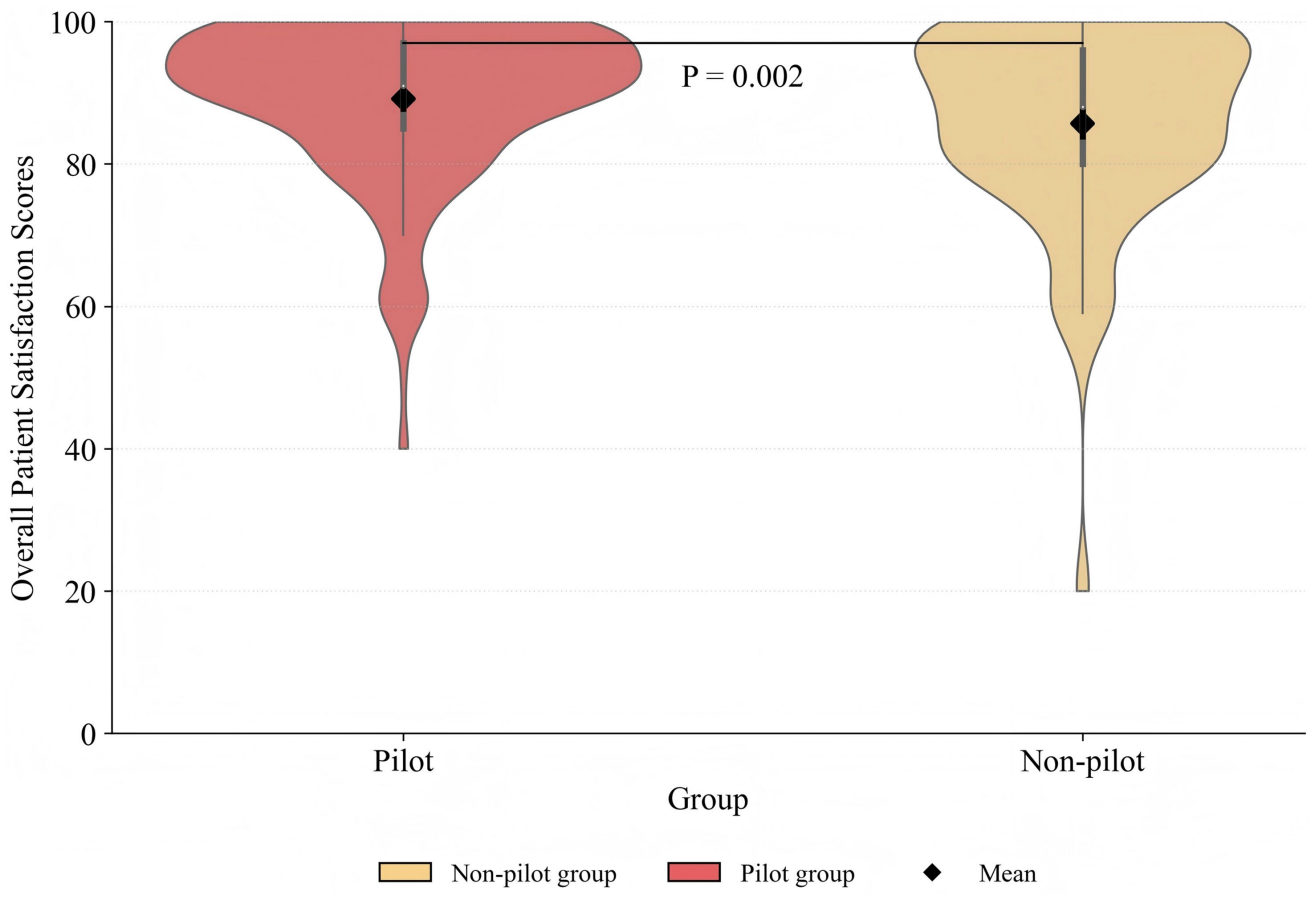
Comparison of overall patient satisfaction scores in pharmaceutical services between the pilot and non-pilot groups.

The distribution of overall patient satisfaction with pharmaceutical services scores across the pilot and non-pilot groups is shown in Figure 1. The violin plot shows that both groups had comparable distributions, with scores heavily concentrated between 80 and 100. The pilot group shows a higher density of values in this range. The range of overall patient satisfaction scores is wider in the non-pilot group, suggesting more variability. The mean score of overall patient satisfaction in the pilot group (89.13 ± 10.99) was higher than that in the non-pilot group (85.67 ± 13.87) (*t* = −3.095, *p* = 0.002). Additionally, higher overall satisfaction scores were observed among males (*t* = 2.037, *p* = 0.042) and among participants whose attending physician held a senior title (*t* = 2.720, *p* = 0.007). Furthermore, individuals with an annual income of CNY 30,001–60,000 (*F* = 6.31, *p* = 0.002), respondents from Guanzhong (*F* = 6.08, *p* = 0.003), and those with a high school diploma or above (*F* = 5.20, *p* = 0.006) also reported higher overall patient satisfaction scores.

### 3.3 Patient satisfaction with pharmaceutical services by dimension

Table 2 presents patient satisfaction with pharmaceutical services across different dimensions, with comparisons between the pilot and non-pilot groups. The percentage of patients satisfied with the promptness of medication receipt was higher in the pilot group than in the non-pilot group (87.32% vs. 74.54%, χ^2^ = 13.245, *p* < 0.001). Regarding pharmacists’ attitude, the percentage of satisfied patients for solving medication issues (88.04% vs. 75.93%, χ^2^ = 12.449, *p* < 0.001) and understanding medical cases (77.90% vs. 64.35%, χ^2^ = 11.018, *p* = 0.001) was higher in the pilot group than in the non-pilot group. The percentage of satisfied patients with the clarity of medication label instructions was 77.54% in the pilot group, which was 13.65 percentage points higher than that in the non-pilot group (63.89%) (χ^2^ = 11.086, *p* = 0.001). The proportion of patients satisfied with the clarity and legibility of medication names was 56.52% in the pilot group and 49.07% in the non-pilot group, with no statistically significant difference observed. Regarding the pharmacy location, cleanliness and acceptability (87.32% vs. 63.89%, χ^2^ = 5.315, *p* = 0.021) and the suitability of working hours (80.80% vs. 71.76%, χ^2^ = 5.560, *p* = 0.018) in the pilot group exhibited higher patient satisfaction percentage than in non-pilot group. In medication teaching, the percentage of satisfied patients in the pilot group was higher than in the non-pilot group except for dosage instruction (*p* < 0.05), including medication reasons (86.23% vs. 76.85%), side effects (92.75% vs. 83.33%), storage (89.13% vs. 81.94%), education time (30.43% vs. 14.35%), and private space (82.61% vs. 74.54%). Notably, while the percentage of satisfied patients with medication education time in the pilot group was 16.05 percentage points higher than that in the non-pilot group, this item still had the lowest patient satisfaction percentage across all items.

**Table 2:** Patient satisfaction with pharmaceutical services by dimension, n (%).

Dimensions	Items	Non-pilot group	Pilot group	χ^2^	*P*
Promptness	Receive medications within a reasonable time				
	Dissatisfied	55(25.46)	35(12.68)	13.245	<0.001
	Satisfied	161(74.54)	241(87.32)		
	Waiting time is acceptable considering the quantity of prescription medication				
	Dissatisfied	18(8.33)	20(7.25)	0.201	0.654
	Satisfied	198(91.67)	256(92.75)		
Attitude	Pharmacist helped me to get the medications				
	Dissatisfied	52(24.07)	47(17.03)	3.742	0.053
	Satisfied	164(75.93)	229(82.97)		
	Pharmacist helped to solve any problem getting the medication				
	Dissatisfied	52(24.07)	33(11.96)	12.449	<0.001
	Satisfied	164(75.93)	243(88.04)		
	Pharmacist answered my questions				
	Dissatisfied	57(26.39)	75(27.17)	0.038	0.845
	Satisfied	159(73.61)	201(72.83)		
	Pharmacist understood the medical case				
	Dissatisfied	77(35.65)	61(22.10)	11.018	0.001
	Satisfied	139(64.35)	215(77.90)		
	Pharmacists treat me with respect				
	Dissatisfied	82(37.96)	101(36.59)	0.097	0.755
	Satisfied	134(62.04)	175(63.41)		
Supply	Medication quantity was sufficient				
	Dissatisfied	41(18.98)	39(14.13)	2.094	0.148
	Satisfied	175(81.02)	237(85.87)		
	All my medications were available in the pharmacy				
	Dissatisfied	37(17.13)	34(12.32)	2.271	0.132
	Satisfied	179(82.87)	242(87.68)		
	Medication name was clear and easy to read				
	Dissatisfied	110(50.93)	120(43.48)	2.700	0.100
	Satisfied	106(49.07)	156(56.52)		
	Medication label/sticker instructions were clear				
	Dissatisfied	78(36.11)	62(22.46)	11.086	0.001
	Satisfied	138(63.89)	214(77.54)		
	Medication appearance and quality was good				
	Dissatisfied	22(10.19)	32(11.59)	0.246	0.620
	Satisfied	194(89.81)	244(88.41)		
Place	The pharmacy was easily found				
	Dissatisfied	52(24.07)	48(17.39)	3.342	0.068
	Satisfied	164(75.93)	228(82.61)		
	The waiting area was comfortable				
	Dissatisfied	53(24.54)	49(17.75)	3.393	0.065
	Satisfied	163(75.46)	227(82.25)		
	The pharmacy area was clean and acceptable				
	Dissatisfied	44(20.37)	35(12.68)	5.315	0.021
	Satisfied	172(79.63)	241(87.32)		
	The pharmacy working hours are suitable to me				
	Dissatisfied	61(28.24)	53(19.20)	5.560	0.018
	Satisfied	155(71.76)	223(80.80)		
Teaching	Pharmacist explained the reason for my medication				
	Dissatisfied	50(23.15)	38(13.77)	7.259	0.007
	Satisfied	166(76.85)	238(86.23)		
	Pharmacist told how to take the correct medication dose				
	Dissatisfied	14(6.48)	9(3.26)	2.820	0.093
	Satisfied	202(93.52)	267(96.74)		
	Pharmacist explained my medication’s possible side effects				
	Dissatisfied	36(16.67)	20(7.25)	10.661	0.001
	Satisfied	180(83.33)	256(92.75)		
	Pharmacist explained how to store my medication				
	Dissatisfied	39(18.06)	30(10.87)	5.189	0.023
	Satisfied	177(81.94)	246(89.13)		
	I had enough time with the pharmacist				
	Dissatisfied	185(85.65)	192(69.57)	17.499	<0.001
	Satisfied	31(14.35)	84(30.43)		
	Pharmacy had a private place for teaching medication use				
	Dissatisfied	55(25.46)	48(17.39)	4.769	0.029
	Satisfied	161(74.54)	228(82.61)		

### 3.4 Determinants of patient satisfaction with pharmaceutical services

Table 3 presents the results of multilevel linear regression models with random intercepts. The implementation of CPS was positively associated with patient satisfaction with pharmaceutical services ( β =3.264, P = 0.010). This result indicates that patients who received pharmaceutical services in hospitals with the CPS had higher satisfaction compared to those in hospitals without this policy. In addition, rural residence was significantly associated with lower satisfaction scores ( β = − 3.307, P = 0.011).

**Table 3:** Association between patient satisfaction with pharmaceutical services and its determinants.

Variables	β	S.E.	*P*	95%CI	
				Lower	Upper
CPS (Ref: Not implemented)					
Implemented	3.264	1.270	0.010	0.774	5.753
Gender (Ref: Male)					
Female	−1.991	1.073	0.063	−4.093	0.111
Age (Ref: ≤ 34 years)					
35 ~ 60	0.558	1.289	0.665	−1.969	3.085
≥ 61	−0.834	1.771	0.638	−4.305	2.637
Income (Ref: ≤30,000 CNY)					
30,001 ~ 60,000	1.666	1.382	0.228	−1.044	4.375
≥60,001	−3.721	2.881	0.197	−9.368	1.926
Marital status (Ref: Unmarried)					
Married	1.828	2.286	0.424	−2.653	6.308
Education level (Ref: Elementary and below)					
Junior high school	−2.693	2.511	0.283	−7.616	2.229
High school and above	−0.092	2.725	0.973	−5.434	5.249
Area(Ref: Southern Shaanxi)					
Northern Shaanxi	2.334	2.068	0.259	−1.719	6.386
Guanzhong	3.642	1.888	0.054	−0.058	7.341
Residence (Ref: Urban)					
Rural	−3.307	1.296	0.011	−5.848	−0.766
Hospital grade (Ref: Tertiary)					
Secondary	−1.801	1.377	0.191	−4.506	0.904
Professional title (Ref: Senior title)					
Non-senior title	−2.117	1.197	0.077	−4.463	0.229
Department (Ref: Internal medicine)					
Surgery	−0.232	1.184	0.844	−2.553	2.088
Others	−1.105	1.432	0.440	−3.911	1.702
Intercept	89.936	4.598	<0.001	80.924	98.949

Loglikelihood = −1913.719, Wald χ^2^(15) = 46.55, P < 0.001, AIC = 3,863.437, BIC = 3,939.010.

## 4 Discussion

As the reform of public hospitals progresses, CPS plays an increasingly active role in promoting rational medicine use and pharmaceutical service quality. To the best of our knowledge, this is the first study to compare patient satisfaction with pharmaceutical services between the pilot and non-pilot public hospitals and to explore the association between implementation of CPS and patient satisfaction. The study had three important findings.

Firstly, overall patient satisfaction with pharmaceutical services was high, and it was significantly higher in pilot hospitals than in non-pilot hospitals. Specifically, the mean satisfaction score in the pilot group (89.13 ± 10.99) was higher than that of the non-pilot group (85.67 ± 13.87). While this absolute difference may appear modest, it should be interpreted in the context of the well-documented ceiling effect typical of patient satisfaction metrics in healthcare settings, where responses tend to cluster at the top of the scale (31). This phenomenon arises from the inherently positive perceptions of essential care services (32). Within such a compressed high-scoring distribution, even marginal improvements can indicate meaningful enhancements in the patient experience and service quality. In the context of CPS, higher patient satisfaction likely serves as a clear mandate for management to redirect resources into patient-centered initiatives, thereby reducing irrational drug expenditure and improving both patient well-being. In addition, the mean satisfaction score in the pilot group was higher than that reported in a Chinese study by Huang et al. (33), which showed a score of 86.19 ± 16.13 in primary care settings. Our score was also higher than those reported in studies from Ethiopia (30.6) (34) and Iran (57) (35). Compared to the systems in Ethiopia, where the director of pharmacy is primarily responsible for managing drug dispensaries (36), the CPS establishes an integrated organizational framework that promotes collaboration among medical staff in China (5). While comparable to roles like the “director of pharmacy” in terms of managerial duties, the Chinese Chief Pharmacist not only retains their foundational identity as a medication expert, but also assumes an executive leadership position in the hospital’s pharmaceutical administration (11, 37). This hybrid role facilitates the implementation of pharmaceutical services, which may contribute to higher patient satisfaction. These comparisons must be interpreted cautiously, as patient satisfaction with pharmaceutical services was measured with different tools and types of hospital services. Nevertheless, the practices and experiences of CPS could provide guidance for hospitals in China and other developing countries wishing to develop pharmaceutical services.

Secondly, although the percentage of satisfied patients with medication education time was lowest, the pilot group still performed better than the non-pilot group. This is likely attributed to the understaffing of pharmacists in Chinese public hospitals. The survey of tertiary hospitals in China (6) found that the average number of pharmacists per 100 beds was 5.6, approximately half that of the United States (24). The shortage of pharmacists, combined with high patient volumes, limits the time pharmacists can devote to individual consultations (38, 39). Consequently, patients often struggle to identify prescribed medications (40, 41), and pharmacists have insufficient opportunity to provide clear verbal or written explanations (42). In the future, innovative approaches such as Telepharmacy could offer a promising direction for expanding the reach and efficiency of pharmacist-led patient education in resource-constrained settings (43, 44). In addition, no significant differences were observed between the groups for items such as “medication name clarity” and “medication appearance and quality.” This is an expected finding, as the characteristics of the medication are predominantly governed by pharmaceutical manufacturers and national drug regulatory standards. Therefore, CPS has a limited impact on improving drug packaging and physical quality.

Thirdly, there was a positive association between the implementation of CPS and patient satisfaction with pharmaceutical services. This association may be explained by the following three hypothesized mechanisms. The first potential mechanism is that establishing an indicator system for rational medication use could improve service efficiency, which might correlate with higher patient satisfaction. Our finding indicated that the pilot hospitals had a higher satisfaction rate regarding the promptness of medication receipt compared to the non-pilot hospitals, which aligns with Yang et al. (10). Furthermore, the satisfaction rate for clear medication label instructions in the pilot hospitals was 13.65 percentage points higher than that in the non-pilot hospitals. This is consistent with Alburikan et al. (45), who suggested that independent access to and understanding of medication information fosters patient confidence, thereby potentially increasing satisfaction. The second proposed mechanism involves the transition of pharmacy departments from a “drug-centered” to a “patient-centered” service model, which is hypothesized to enhance the quality and accessibility of pharmacist-patient interactions. We speculate that this improved interaction quality would be associated with higher patient satisfaction. In the context of CPS, this shift may be supported by professional training and optimization of pharmacy environments. Professional training equips pharmacists with expanded pharmacotherapy knowledge, which may improve their effectiveness in patient counseling (5, 46). As Aziz et al. (47) indicated, such improvements in service quality directly fulfill patients’ needs for competent and attentive care, thereby boosting satisfaction. Our findings also offer partial support for this hypothesis. Pilot hospitals had a higher patient satisfaction rate than non-pilot hospitals across several dimensions, including pharmacists’ attitude toward resolving medication issues, understanding medical cases, and providing clear explanations of medication rationale, side effects, and storage requirements. Furthermore, the cleanliness and acceptability of pharmacy areas, suitable pharmacy working hours, and private teaching spaces were associated with higher satisfaction. These findings are common with previous studies. Suleiman et al. (48) and Altarifi et al. (49) suggested that pharmacy location, cleanliness, and service hours were key factors contributing to improved patient satisfaction with pharmaceutical services. Notably, availability of private counseling areas serves as an essential component of pharmaceutical service quality and plays a key role in improving patient satisfaction (15). The third hypothesized mechanism is that establishing an interdisciplinary team to monitor and review key medicines might improve prescribing rationality and medication safety. It is plausible that these improvements are likely associated with greater patient satisfaction. Ma et al. (5) noted that with the implementation of CPS, pharmacists could actively participate in multidisciplinary teams to optimize antibacterial drug selection and adjust drug dosages, which could reduce irrational prescribing. Based on the above findings, this study suggests that the government should make efforts to expand the implementation of the CPS to non-pilot hospitals to enhance pharmaceutical services. The pilot hospitals should prioritize resolving the time constraints on individualized patient counseling and medication education. These supplementary measures could be adopted, such as optimizing pharmacy physical spaces and advancing targeted pharmacist training.

Fourthly, our findings indicated that satisfaction among rural patients was significantly lower than that of their urban counterparts, a result consistent with previous studies (50, 51). Disparities in socioeconomic conditions and healthcare resources between rural and urban areas may result in different patient perceptions during pharmaceutical services utilization (52). Additionally, rural patients may encounter greater barriers related to convenience and access to pharmaceutical information, which may further constrain their satisfaction with such services (53). It is suggested that there is a need to improve rural healthcare infrastructure and staffing to enhance the quality and accessibility of rural pharmaceutical services.

This study has several limitations. Firstly, as a cross-sectional survey, this study is limited to identifying associations and cannot establish causality between implementation of CPS and patient satisfaction. It is unable to capture any long-term dynamics. Therefore, longitudinal or experimental studies are required to investigate potential causal relationships, analyze the evolution of satisfaction, and identify the determinants that drive its improvement over time. Secondly, several measurement biases may be present. Although patients were surveyed immediately after leaving the pharmacy, the potential for recall bias remains. The self-reported satisfaction is subject to ceiling effects that may compress variance and reduce the discernibility of group differences. Furthermore, social desirability bias may have been introduced as participants were surveyed on-site and might have provided more favorable responses than they actually held. Sampling based on inpatient dispensing records may introduce selection bias by omitting patients who were dissatisfied and disengaged. Thirdly, although hospital grade was considered, other hospital-level confounders such as staffing ratios and institutional resources were not controlled. Fourthly, multiple chi-square tests were conducted without correction for multiple comparisons, which may increase the risk of type I error. Although these comparisons were performed primarily among control variables, future studies should apply appropriate statistical adjustments to enhance the robustness of the findings. Finally, the study sample was drawn exclusively from Shaanxi Province, China, which may be affected by local healthcare systems, culture, and socio-economic factors. This regional concentration may limit the generalizability of the conclusions.

## 5 Conclusion

Patient satisfaction with pharmaceutical services was higher in pilot hospitals than in non-pilot hospitals. The implementation of CPS was positively associated with this satisfaction. Although satisfaction regarding medication education time remained the lowest among all items, the pilot group still outperformed the non-pilot group in this aspect. These findings suggest that expanding CPS could be a beneficial strategy for enhancing patient satisfaction. Pilot hospitals should consider prioritize addressing time constraints in individualized patient counseling. As potential supportive measures, optimizing pharmacy physical spaces and advancing targeted pharmacist training may also be considered.

## Data Availability

The original contributions presented in the study are included in the article/supplementary material, further inquiries can be directed to the corresponding author.
